# Responder Analysis of a Multicomponent Non-Pharmacological Intervention (MAKS) for People With Cognitive Impairment in the German Day-Care Study (DeTaMAKS)

**DOI:** 10.3389/fpsyt.2019.00587

**Published:** 2019-08-21

**Authors:** Katharina Luttenberger, Elmar Graessel, Elisa-Marie Behrndt, Dominik Özbe, Carolin Donath, Jennifer Scheel

**Affiliations:** Center for Health Services Research in Medicine, Department of Psychiatry and Psychotherapy, University Hospital Erlangen, Friedrich-Alexander-University Erlangen-Nürnberg, Erlangen, Germany

**Keywords:** dementia, cognitive impairment, multicomponent intervention, non-pharmacological intervention, responder analysis

## Abstract

**Background:** Multicomponent non-pharmacological therapies have been shown to be effective at reducing cognitive symptoms and slowing deterioration in abilities to perform activities of daily living (ADL) in individuals with cognitive impairment. However, little is known about response rates and predictors of response.

**Methods:** We used data from the German day-care study (DeTaMAKS; De = dementia, Ta = Tagespflege/day-care, M = motor stimulation, A = activities of daily living stimulation, K = k/cognitive stimulation, S = social stimulation; n = 362), which was based on a cluster-randomized trial of the non-pharmacological, multicomponent, anti-dementia MAKS therapy for people with cognitive impairment in day-care centers. We investigated response (defined as improvement or no deterioration) for three different response criteria: cognition via Mini-Mental State Examination (MMSE) score, ADL via Erlangen Test of Activities of Daily Living in Persons with Mild Dementia or Mild Cognitive Impairment (ETAM) score, and behavioral and psychological symptoms of dementia (BPSD) via Neuropsychiatric Inventory Questionnaire (NPI-Q) score. In addition, we calculated the number needed to treat (NTT) and response rates according to net gain analyses.

**Results:** For all three criteria, the response rates were higher in the intervention group than in the control group (chi^2^ test: *p* = *0.058* to *p* = *0.003*). Compared with non-responders, responders according to cognition had higher ETAM scores (= better ADL abilities) at baseline; responders according to ADL had lower ETAM scores (= poorer ADL abilities) at baseline; and responders according to BPSD had higher NPI-Q scores (= more BPSD) at baseline. Classification rates based on these predictors ranged from 60.6 to 68.3%.

**Discussion:** The response rates to the non-pharmacological MAKS therapy were greater than those reported for anti-dementia drugs. There were only a few differences between responders and non-responders. Because of the low classification rates, these variables had only a small impact on response predictions. Therefore, there are no empirically substantiated selection criteria for the application of MAKS therapy in facilities.

**Clinical Trial Registration:** www.ClinicalTrials.gov, identifier ISRCTN16412551.

## Introduction

According to the World Health Organization (WHO) (1), the total number of individuals with dementia worldwide is estimated at 50 million and may triple within the next 30 years. Dementia is already the second leading cause of need for care among older people, and it has a large impact on the psychological well-being of caregivers and families (2). There have also been dramatic increases in the costs of medical care and the societal burdens associated with this disease (3).

In cholinesterase inhibitor (CEI) trials, intervention groups (IGs) have shown around 14 to 37% more responders on cognitive outcomes than placebo groups, depending on the drug, severity of dementia, and response criterion (4). Yet, a recent cohort analysis called into question the long-term effects by showing that initial improvement on the Mini-Mental State Examination (MMSE) seemed to vanish after about 1 year of treatment (5). In addition, treatment with CEIs was associated with adverse effects such as bradycardia or syncope (6). In a recent responder analysis for patients with mild to moderate Alzheimer’s disease treated with Donepezil, differences in the response rates of the IG versus the placebo group of 12 to 16% were found on cognitive outcomes (7).

Medical guidelines on the treatment of dementia from the NICE Institute (8) as well as its German equivalent (9) recommend the use of non-pharmacological therapies such as cognitive stimulation or the occupational training of activities of daily living (ADL); both of which are on a par with pharmacological treatment via CEIs. Non-pharmacological therapies addressing cognition, ADL, and BPSD have been shown to be effective in a variety of trials. For example, cognitive stimulation showed beneficial effects on cognition in addition to medication (10). Physical activity led to a reduction in BPSD (11) and showed promising results for the improvement of ADL (12). However, in a recent review, the quality of these studies was regarded as mostly low (13). In recent research, non-pharmacological multicomponent therapies seemed to be more effective than one-component therapies because they had positive effects on more than one outcome domain (14, 15). In two high-quality-randomized controlled trials, MAKS therapy, a multicomponent group therapy for people with mild cognitive impairment and mild to moderate dementia (components: motor stimulation, M; activities of daily living stimulation, A: k/cognitive stimulation, K; social stimulation, S), was shown to be effective in maintaining the cognitive and ADL abilities of elderly individuals with cognitive impairments, living either in nursing homes (16, 17) or in the community (18). Yet, to our knowledge, responder analyses for non-pharmacological therapies targeting cognitive impairment (mild cognitive impairment and mild to moderate dementia) have not been published, whereas several studies have explored responses to pharmacological therapies (19).

A response is usually defined as a stabilization or improvement in dementia symptoms. According to ICD 10 criteria (20), no response is defined as the further progression of dementia-associated symptoms: a) cognitive decline, b) a deterioration in activities of daily living, and c) an increase in behavioral psychological symptoms of dementia (BPSD).

Several variables might influence or even predict response: whether or not a person responds to an intervention might depend on several factors regarding the person with dementia (PWD) himself/herself and factors regarding the environment of the PWD (e.g., a caregiving relative). Possible predictors of response referring directly to the PWD might be diseases involving physical impairment of the upper extremities (which might particularly influence component M “motor stimulation”) (21), sex (which might particularly influence component A “activities of daily living stimulation,” especially in this generation) (22), severity of cognitive impairment (which might particularly influence component K “k/cognitive stimulation,” as cognitive stimulation is recommended for the earlier stages of dementia) (9), agreeableness (which might particularly influence component S “social stimulation”) (23), depressive symptoms [which might influence all four components M, A, K, and S, see e.g., Watts et al. (24)], and age. Possible predictors of response referring to the PWD’s environment might be a stimulating environment outside of day care, living alone/living with the caregiving relative, abusive behavior at home, and the burden and age of the caregiver.

The aim of this paper is to perform a responder analysis of MAKS therapy with the outcome criteria cognition, ADL, and BPSD and to identify the predictors of response. This analysis should allow us to identify treatment responders as well as the most promising treatments. It may also help to improve their cost-effectiveness.

Research questions:

1. How many responders are in the intervention group (IG) versus the control group (CG) for the different response criteria?
2. Do any of the variables predict response—analyzed separately for the different response criteria?

## Materials and Methods

### Design

The present analyses were computed on the per protocol sample from the German day-care study and included 362 day-care users. The German day-care study (DeTaMAKS) is a cluster-randomized controlled trial, which was conducted in 32 day-care centers throughout Germany (25). The day-care centers were randomly assigned to either the IG or the CG, stratified by region. In the IG (16 day-care centers), MAKS therapy, a multimodal non-pharmacological training for people with cognitive impairment, was offered once a day. Participants received MAKS therapy each day they visited the day-care center (between 1 and 5 days per week). In the CG (16 day-care centers), participants received “care as usual,” which included offers of stimulating treatments besides MAKS to all participants in the day-care center (active control group) and treatment with anti-dementia drugs (about 30% of the whole sample). The study design is described in detail in Behrndt et al. (25).

### Intervention

MAKS therapy is a structured, multimodal, non-pharmacological therapy that addresses the following components: (psycho) motor capabilities (M), ADL abilities (A), cognitive functioning (K), and social components (S). The therapy is manualized, and a unique program is offered every day, each addressing all four components. These include, for example, discussions of personal topics with seasonal references (social), table football (motor), crafting a bird box (ADL), and playing a specially adapted version of “Who Wants to be a Millionaire” projected on a screen (cognition). Qualified staff members working in the day-care centers were trained for 2 days to use and apply the materials in the manual. They subsequently offered a 2-h MAKS therapy session every day. For a detailed description of the intervention and its implementation, see Behrndt et al. (25) and Straubmeier et al. (18).

Implementation fidelity was ensured by 1) a structured 2-day training for the persons conducting MAKS in the day-care center, 2) the MAKS instruction manual with detailed instructions how to perform the intervention, 3) documentation by the MAKS therapists in the day-care centers to monitor the intensity and quality of the MAKS therapy in the intervention group, and 4) monitoring by the study headquarters staff in the day-care centers during the first 6 months in a random sample of four day-care centers (investigating treatment adherence/conduct of MAKS therapy, quality of the documentation, and attendance of the study participants).

### Data Collection

Data were collected from all participants at baseline and after the intervention had run for 6 months. Data were obtained from the day-care users in three different ways. First, the Mini-Mental State Examination (MMSE, cognitive performance) and the Erlangen Test of Activities of Daily Living in Persons with Mild Dementia or Mild Cognitive Impairment (ETAM, ADL performance) were assessed directly by trained staff members who worked at the day-care centers but were not involved in the MAKS therapy. Second, proxy ratings of BPSD were given by the primary caregiver via telephone interviews conducted by trained researchers from the study headquarters. Third, data on medications were obtained directly from the documentation of the day-care centers.

### Instruments

#### Predictor Variables (Assessed at Baseline): PWD Data

**Charlson Comorbidity Index (** 26,**):** The Charlson Comorbidity Index (26) was updated by Quan et al. (27) and is used to calculate the effect of any previous medical diagnoses on the mortality rate. Thus, it assigns weights to comorbidities according to their severity. The 1-year mortality increases from 12% (index = 0) to 85% (index ≥ 5) as the score increases (26).
**Care level:** The care level describes the extent of independence in daily life and ranges from none (fully independent) to level 5 (most severe impairment to independence with special demands placed on nursing care). In Germany, this scale is used as a reference point to classify a person’s entitlement to care services from the German long-term care insurance (German system of statutory long-term care funds).
**Anti-dementia drugs:** Anti-dementia drugs (memantine and cholinesterase inhibitors) were assessed with the documentation system used by the day-care centers.
**Depressive symptoms:** Depressive symptoms were assessed with the depression item from the Neuropsychiatric Inventory Questionnaire (NPI-Q) (28). For more information, see the description of the NPI-Q in the outcome section.
**CNS depressant score:** The central nervous system (CNS) depressant score was formed in the following way: all medications were independently rated by two experts—both professors of clinical pharmacology at the University of Erlangen-Nürnberg—on a scale comprised of “very CNS depressant” (−2), “CNS depressant” (−1), “neutral” (0), “CNS activating” (+1), and “very CNS activating” (+2). The scores from a PWD’s medications were summed and formed the PWD’s CNS depressant score.
**Orthopedic and neurological diseases:** Orthopedic diseases (e.g., arthrosis, polyarthritis, gout; included ICD codes [not all present in the sample]: M06, M10, M15, M16, M17, M19, M20, M25, M41, M42, M43, M47, M48, M51, M54, M62, M79, M80, M81, M93) and neurological diseases (e.g., Parkinson’s disease, multiple sclerosis, stroke; included ICD codes [not all present in the sample]: G20, G21, G25, G35, G36, G40, G41, G43, G45, G50, G62, G63, G70, G71, G72, G81, G83, G90, G91, G93, I63, I64) were assessed with the documentation system used by the day-care centers.

#### Predictor Variables (Assessed at Baseline): Caregiver Variables

**Burden scale for family caregivers—short version (BSFC-s) (** 29 **):** The BSFC-s is used to assess the subjective burden of informal caregivers. The 10 items are rated on a scale ranging from 0 (strongly disagree) to 3 (strongly agree). The total score ranges from 0 to 30 points, with higher values indicating greater burden.
**Positive aspects of caregiving:** the Berlin Inventory of Caregivers’ Burden with Dementia Patients (Berliner Inventar zur Angehörigenbelastung—Demenz, BIZA-D) (30) contains a 5-item subscale for “benefits” rated on a five-point Likert scale (0–4). The sum score ranges from 0 to 20 points, with higher values indicating that the caregiver is experiencing the more positive aspects of caregiving.

The age of the caregiving relative, the degree of kinship to the PWD, and whether the caregiver lived in the same house with the PWD (shared or separate households) were also assessed via telephone interviews with the primary caregiver.

#### Outcome Variables (Assessed at Baseline and After 6 Months in the Intervention): Dementia Symptoms

**Mini-Mental State Examination (MMSE) (** 31 **):** The MMSE measures five areas of cognitive functioning: orientation, registration, attention and calculation, recall, and language. The score ranges from 0 to 30 points, with higher scores representing better cognitive performance. This is the most often used screening test for dementia worldwide and thus provides good comparability with other studies. Furthermore, the MMSE is suitable for the assessment of cognitive function in longitudinal studies when basic information on cognition is sufficient (32).
**Erlangen Test of Activities of Daily Living in Persons with Mild Dementia or Mild Cognitive Impairment (ETAM) (** 33 **):** The ETAM is a short performance test for the assessment of the ability to perform ADL in people with mild dementia or mild cognitive impairment. The score ranges from 0 to 30 points, with higher values showing better abilities to perform ADL (33).
**Neuropsychiatric Inventory Questionnaire (NPI-Q) (** 28 **):** The NPI-Q is an observer rating scale for the evaluation of neuropsychiatric symptoms by the informal caregiver covering the 12 symptom areas: delusions, hallucinations, agitation/aggression, depression/dysphoria, anxiety, elation/euphoria, apathy/indifference, disinhibition, irritability/lability, motor disturbance, night-time behaviors, and appetite/eating. In the DeTaMAKS project, the NPI-Q symptoms were assessed only dichotomously (presence or absence of a symptom). The sum score therefore ranges from 0 to 12 points. Here, the NPI-Q score was used as a proxy for BPSD.

### Definition of Response

Because a deterioration of three MMSE points per year can be expected in untreated dementia patients (34,), we formed three groups that reflected different levels of MMSE change: responders (improvement or no deterioration in 6 months), expected deteriorators (deterioration ≤1.5 MMSE points in 6 months), and pronounced deteriorators (deterioration >1.5 MMSE points in 6 months).

Furthermore, three response criteria were formed on the basis of the ICD criteria for dementia: a) cognition (MMSE score), b) ADL (ETAM score), and c) BPSD (NPI-Q score as the number of BPSD items). For all three criteria, response was defined as improvement or no deterioration according to the National Institute for Health and Care Excellence (NICE) guideline (4).

### Statistical Analyses

The means, standard deviations, minima, maxima, and frequencies (when appropriate) were given for all variables to provide an overview.

Group differences regarding non-normally distributed variables were calculated using Mann–Whitney U-tests. The variables that differed between responders and non-responders were used as predictors in binary logistic regression analyses. These were calculated to analyze the classification rates for the three response criteria. The baseline scores of the outcome variables are also used as potential predictor variables to control for baseline differences.

Group differences regarding the numbers of responders in the IG and in the CG were calculated using chi^2^ tests (according to Fisher). In addition, a net gain analysis (36) was computed for the three MMSE groups. The net gain was defined as (% responders_IG_—% responders_CG_—[% deteriorators_IG_—% deteriorators_CG_]) (36). The number needed to treat (NNT; how many patients must undergo MAKS therapy for one more patient to remain stable/improve) was calculated for all response criteria.

## Results

### Description of the Sample

Sixty one percent of the participants were women, and the mean age of the sample was 81.3 years (*SD* = 7.5). Participants achieved a mean MMSE value of 19.6 (*SD* = 4.8) with cognitive impairment ranging from mild cognitive impairment to mild and moderate dementia. The rate of people with mild cognitive impairment [defined as MMSE ≥24 and Montreal Cognitive Assessment (MoCA) ≤22 (18)] did not differ between intervention and control group (chi², Fisher’s exact test, p = 0.444). Around one third of all participants were treated with anti-dementia drugs (CEIs or memantine). Detailed descriptive data of the IG, the CG, and the whole sample are presented in **Table 1**. There were no significant differences between the IG and CG on the baseline data except the CNS depressant score (CG-PWDs had a higher CNS depressant score than IG-PWDs) and the variable reflecting whether the caregiver lived in the same house with the PWD (CG-PWDs were more likely to live in the same house with their caregiver).

**Table 1:** Baseline characteristics of the sample.

					Whole sample (N = 362)	Intervention group (n = 208)	Control group (n = 154)	p for group differences between intervention and control group
PWD variables at baseline	Age (in years)			M ± SD	81.3 ± 7.5 (46–99)	81.5 ± 7.5 (53–99)	81.1 ± 7.5 (46–93)	0.701^a^
	Sex (female)			% (n)	61.0% (n = 221)	61.1% (n = 127)	61.0% (n = 94)	>0.999^b^
	Education (years of school attendance)		<8 years 8–9 years 9–10 years 12–13 years university	% (n)	6.4% (n = 23) 70.2% (n = 254) 12.7% (n = 46) 5.0% (n = 18) 5.8% (n = 21)	6.7% (n = 14) 69.7% (n = 145) 12.0% (n = 25) 4.8% (n = 10) 6.7% (n = 14)	5.8% (n = 9) 70.8% (n = 109) 13.6% (n = 21) 5.2% (n = 8) 4.5% (n = 7)	0.910^b^
	Dementia symptoms	Cognition (MMSE)		M ± SD	19.6 ± 4.8 (10–30)	19.8 ± 4.8 (10–30)	19.3 ± 4.8 (10–30)	0.267^a^
		ADL (ETAM)		M ± SD	17.6 ± 7.2 (0–30)	17.9 ± 6.9 (0–30)	17.1 ± 7.5 (2–30)	0.340^a^
		BPSD (NPI-Q)		M ± SD	5.3 ± 2.7 (0–11)	5.2 ± 2.7 (0–11)	5.4 ± 2.7 (0–11)	0.572^a^
	Medical variables	Charlson Comorbidity Index		M ± SD	2.2 ± 1.6 (0–8)	2.3 ± 1.6 (0–8)	2.1 ± 1.6 (0–7)	0.425n.s.^a^
		Anti-dementia drugs^1^ (yes)		% (n)	28.2% (n = 102)	29.8% (n = 62)	26% (n = 40)	0.479^b^
		Care level		M ± SD	2.9 ± 0.8 (1–5)	2.9 ± 0.8 (1–5)	3.0 ± 0.9 (1–5)	0.362^a^
		Depression (NPI-Q)		M ± SD	0.6 ± 0.5 (0–1)	0.6 ± 0.5 (0–1)	0.6 ± 0.5 (0–1)	0.721^a^
		CNS depressant score		M ± SD	−1.1 ± 1.6 (−10–1)	−1.0 ± 1.5 (−10–1)	−1.4 ± 1.7 (−8–1)	**0.019** **^a^**
		Orthopedic diseases		% (n)	27.9% (n = 101)	28.8% (n = 60)	26.6% (n = 41)	0.722^b^
		Neurological diseases		% (n)	24.3% (n = 88)	21.6% (n = 45)	27.9 (n = 43)%	0.175^b^
Caregiver variables at baseline	Age (in years)			M ± SD	59.5 ± 11.4 (25–87)	59.6 ± 11.6 (27–87)	59.3 ± 11.2 (25–86)	0.801^a^
	Sex (female)			% (n)	74.6% (n = 270)	73.1% (n = 152)	76.6% (n = 118)	0.466^b^
	Education (years of school attendance)		<8 years 8–9 years 9–10 years 12–13 years University	% (n)	1.4% (n = 5) 39.2% (n = 142) 35.9% (n = 130) 12.2% (n = 44) 11.3% (n = 41)	2.4% (n = 5) 38.5% (n = 80) 33.7% (n = 70) 13.9% (n = 29) 11.5% (n = 24)	0.0% (n = 0) 40.3% (n = 62) 39.0% (n = 60) 9.7% (n = 15) 11.0% (n = 17)	0.229^b^
	Burden (BSFC-s)			M ± SD	12.5 ± 8.0 (0–30)	12.0 ± 8.2 (0–29)	13.2 ± 7.6 (0–30)	0.097^a^
	Benefits (BIZA-D)			M ± SD	12.6 ± 5.1 (0–20)	12.5 ± 4.9 (0–20)	12.6 ± 5.4 (0–20)	0.756^a^
	Degree of kinship to the PWD	Spouse		% (n)	30.4% (n = 97)	30.0% (n = 54)	30.9% (n = 43)	0.756^b^
		Parent		% (n)	58.9% (n = 188)	59.4% (n = 107)	58.3% (n = 81)	
		Parent-in-law		% (n)	6.6% (n = 21)	5.6% (n = 10)	7.9% (n = 11)	
		Other relative		% (n)	3.5% (n = 11)	4.4% (n = 8)	2.2% (n = 3)	
		Neighbor/friend		% (n)	0.6% (n = 2)	0.6% (n = 1)	0.7% (n = 1)	
	Living in the same house with the PWD (yes)			% (n)	61.0% (n = 221)	56.2% (n = 117)	67.5% (n = 104)	**0.038** **^b^**

^a^Mann-Whitney U-test; ^b^Chi², Fisher’s exact test; ^1^CEIs or memantine.

ADL, Activities of daily living; BIZA-D, The Berlin Inventory of caregivers’ burden with dementia patients (Berliner Inventar zur Angehörigenbelastung – Demenz); BSFC-s, Burden scale for family caregivers – short version; CNS, Central vervous system; ETAM, Erlangen test of activities of daily living in persons with mild dementia or mild cognitive impairment; MMSE, Mini-mental state examination; NPI-Q, Neuropsychiatric inventory questionnaire.

### Comparison of the IG and CG on Expected MMSE Change

For the CG, the mean MMSE change was a deterioration (−0.9 MMSE points), whereas for the IG, the mean MMSE change was a very slight improvement (+0.1 MMSE points). This difference was significant (Mann–Whitney U-test, *p* = *0.008*). For the three MMSE change groups, the following results emerged: in the IG (versus the CG), the percentage of responders was significantly higher, the percentage of pronounced deteriorators was significantly lower, and the percentage of expected deteriorators was similar (see **Table 2** for details). The net gain analysis showed an advantage of 28% for the IG.

**Table 2:** Numbers of responders, expected deteriorators, and pronounced deteriorators regarding MMSE.

	Intervention group (n = 208)	Control group (n = 154)	Delta of the percentage of responders	p (Chi^2^ test, Fisher’s exact test)
Responders (improvement or no deterioration)	58% (n = 121)	42% (n = 65)	16%	**0.003**
Expected deteriorators (deterioration ≤1.5 MMSE points)	13% (n = 26)	17% (n = 26)	4%	0.289
Pronounced deteriorators (deterioration >1.5 MMSE points)	29% (n = 61)	41% (n = 63)	12%	**0.025**

MMSE, Mini-mental state examination; NPI-Q, Neuropsychiatric inventory questionnaire.

### Comparison of IG and CG Regarding the Response Criteria Cognition, ADL, and BPSD

For all three response criteria, the percentage of responders was higher in the IG compared with the CG. This difference was significant for cognition and ADL, but there was only a trend toward significance for BPSD (see **Table 3**). The NNT was 7 for the criteria cognition and ADL and 10 for BPSD.

**Table 3:** Response to the MAKS intervention.

Response criterion	Percentage of responders		Delta of the percentage of responders	p (Chi^2^ test, Fisher’s exact test)
	Intervention group (n=208)	Control group (n=154)		
Cognition	58% (n=121)	42% (n=65)	16%	**0.003**
ADL	61% (n=126)	45% (n=70)	16%	**0.005**
BPSD	69% (n=144)	59% (n=91)	10%	0.058

ADL, activities of daily living; BPSD, behavioral and psychological symptoms of dementia.

### Comparison of Responders and Non-Responders in the IG for the Response Criteria Cognition, ADL, and BPSD

For each of the three response criteria, we found at least one significant difference between responders and non-responders (see **Table 4**):

- Responders according to the cognition criterion had higher ETAM scores at baseline than non-responders (19.2 versus 16.0 points), and their caregivers` education was higher.
- Responders according to the ADL criterion had lower ETAM scores at baseline than non-responders (16.4 *versus* 20.2 points).
- Responders according to the BPSD criterion had higher NPI-Q scores at baseline than non-responders (5.5 *versus* 4.4 points).

**Table 4:** Comparison of responders and non-responders in the IG for the response criteria “cognition,” “ADL,” and “BPSD.”

			p for group differences between responders and non-responders		
			Cognition (MMSE)	ADL (ETAM)	BPSD (NPI-Q)
PWD variables at baseline	Age (in years)		0.882^a^	0.409^a^	0.586^a^
	Sex (female)		>0.999^b^	>0.999^b^	0.541^b^
	Education (years of school attendance)	<8 years 8–9 years 9–10 years 12–13 years University	0.280^b^	0.359^b^	0.872^b^
	Dementia symptoms	Cognition (MMSE)	0.190^a^	0.864^a^	0.427^a^
		ADL (ETAM)	**0.004** **^a^**	**<0.001** **^a^**	0.353^a^
		BPSD (NPI-Q)	0.636^a^	0.087^a^	**0.005** **^a^**
	Medical variables	Charlson Comorbidity Index	0.371^a^	0.836^a^	0.641^a^
		Anti-dementia drugs^1^ (yes)	0.761^b^	0.757^b^	0.517^b^
		Care level	0.639^a^	0.270^a^	0.845^a^
		Depression (NPI-Q)	0.949^a^	0.551^a^	0.675^a^
		CNS depressant score	0.342^a^	0.202^a^	0.696^a^
		Orthopedic diseases	0.539^b^	>0.999^b^	0.508^b^
		Neurological diseases	0.734^b^	0.608^b^	>0.999^b^
Caregiver variables at baseline	Age (in years)		0.523^a^	0.457^a^	0.233^a^
	Sex (female)		0.637^b^	0.263^b^	0.737^b^
	Education (years of school attendance)	<8 years 8–9 years 9–10 years 12–13 years University	**0.021** **^b^**	0.719^b^	0.905^b^
	Burden (BSFC-s)		0.205^a^	0.635^a^	0.085^a^
	Benefits (BIZA-D)		0.767^a^	0.845^a^	0.209^a^
	Degree of kinship to the PWD		0.931^b^	0.871^b^	0.380^b^
	Living in the same house with the PWD		0.962^b^	0.190^b^	0.308^b^

^a^Mann-Whitney U-test; ^b^Chi², Fisher’s exact test; ^1^CEIs or memantine.

ADL, Activities of daily living; BIZA-D, The Berlin inventory of caregivers’ burden with dementia patients (Berliner Inventar zur Angehörigenbelastung – Demenz); BSFC-s, Burden Scale for Family Caregivers – short version, CNS, central vervous system; ETAM, Erlangen test of activities of daily living in persons with mild dementia or mild cognitive impairment; MMSE, Mini-mental state examination; NPI-Q, Neuropsychiatric inventory questionnaire.

The mean changes in the three criteria were improvements (MMSE score: M = 0.1, SD = 3.8, range: −13 to 15; ETAM score: M = 0.3, SD = 4.0, range: −11 to 12; NPI score: M = 0.1, SD = 1.8, range: −6 to 7).

The rate of people with mild cognitive impairment did not differ between responders and non-responders according to the cognition criterion (chi², Fisher’s exact test, p = 0.328), the ADL criterion (chi², Fisher’s exact test, p = 0.869), and the BPSD criterion (chi², Fisher’s exact test, p = 0.292).

Binary logistic regression analyses with the variables that differed significantly between responders and non-responders as predictors and response *versus* non-response as the outcome variable were significant for all three response criteria. The explained variance ranged from 5.5% (BPSD) to 10.1% (ADL) to 10.6% (cognition). The rates of correct classification varied from 63.9% (cognition) to 64.9% (ADL) to 68.3% (BPSD).

## Discussion

The treatment of dementia remains a great challenge. To optimize health care, it is important to develop effective treatment methods, to evaluate them properly, and to identify groups of PWDs that might profit the most. In this paper, we conducted a responder analysis for the previously evaluated non-pharmacological multicomponent MAKS therapy for individuals with cognitive impairments in day-care centers. We analyzed several factors regarding the PWD, the caregiver, and the care situation that we expected that might influence response.

Our main findings were as follows: 1) for the response criteria cognition and ADL, there were more responders in the IG compared with the CG. 2) For the cognition criterion, responders had higher baseline scores regarding activities of daily living than non-responders, but it was the opposite for the ADL criterion. 3) For the BPSD criterion, responders had higher baseline scores regarding BPSD than non-responders. 4) Because of the low classification rates, these variables had only a small impact on the prediction of response.

### Superiority of the IG Compared With the CG

As expected on the basis of earlier studies (16–18), we found that the MAKS intervention was superior to the active control group—all were offered stimulating interventions besides MAKS in their day-care centers (“care as usual”). The mean MMSE score declined for the CG and remained constant for the IG. A deterioration of three MMSE points per year can be expected in untreated dementia patients (34,) because dementia is a progressive disease. Thus, the deterioration of one MMSE point during the 6-month period in the CG was nearly as high as it would be expected. The superiority of the IG was not only due to significantly higher response rates but also to significantly lower rates of pronounced deteriorators compared with the CG. This underlines the special meaning of non-pharmacological interventions such as MAKS, which is designed not only to improve cognition but also to delay the deterioration that is to be expected from the progression of the disease.

Depending on the criterion, we found response rates ranging from 58 to 69% in the IG. The response rate of 58% for the cognition criterion in our study was slightly higher than the response rate of 53.7% (for stabilization or improvement on the MMSE) after 6 months of cholinesterase-inhibitors (CEI) treatment reported by Droogmsa et al. (37).

Differences in the response rates between the IG and CG ranged from 12 to 16% (depending on the criterion), with NNT values ranging from 7 to 10, respectively. Although it is very difficult to compare response rates across different studies because of the great variety of response criteria, MAKS therapy seems to be at least as effective as CEIs. Burns et al. (7), for example, found similar differences in the response rate of 16% between the IG (donepezil) and CG for cognition measured with the MMSE. Yet, it must be noted, that both in the IG and the CG of our study, almost one third of each group received anti-dementia drugs.

### Differences Between Responders and Non-Responders in the IG as Predictors of Response

For all three response criteria (cognition, ADL, BPSD), we did not find differences between responders and non-responders in the IG regarding the MMSE and education at baseline. MAKS seems to be suitable for all included levels of cognitive impairment (mild cognitive impairment, mild dementia, and moderate dementia) and education levels.

Furthermore, MAKS seems to be suitable for all ADL ability levels that were included in the study. Individuals with different ADL abilities improved on different criteria—those with better ADL abilities had a better chance of improving on the cognition criterion (MMSE), and those with lower ADL abilities had a better chance of improving on the ADL criterion (ETAM) itself. Hence, low ADL abilities do not imply that change is no longer possible, but improvement may happen.

For response on the BPSD criterion, the BPSD score at baseline is important. When defined by the BPSD criterion, responders had a mean NPI-Q score slightly above the mean of the whole IG sample, whereas non-responders’ mean NPI-Q score was almost one point below. There might be a minimum number of BPSD symptoms that render improvement visible on statistical tests (ceiling effect).

However, using these variables to make predictions is limited. The baseline variables that differed between responders and non-responders (the ETAM scores for cognition and ADL; the NPI-Q score for BPSD) displayed a tendency but are not suitable for making valid predictions because only less than 70% of the responders could be classified correctly with each of them.

In most of the responder analyses using CEIs, no significant predictors of response (38) were found. If predictors were found in CEI studies, they were medical factors such as concomitant diseases (5, 19). We did not find that concomitant diseases were associated with response on any criterion, not when measured with the Charlson Comorbidity Index or as particular comorbidities such as orthopedic or neurological diseases. All variables associated with response were instead related to dementia symptoms (ADL and BPSD). Because CEI studies usually focus only on the PWD, we also involved the informal caregiver variables in our study as additional potential predictors. However, we also did not find that any of them were predictive of response.

### Factors Regarding the Environment of the PWD

Of the factors pertaining to the environment of the PWD (e.g., living alone or living with the caregiving relative, burden, age, sex, or education of the caregiver), only caregivers` education significantly influenced PWDs` response to the multicomponent non-pharmacological MAKS intervention. The caregivers` education level might be a proxy for a stimulating environment beside day care for the PWD. Caregivers with high education might foster cognitive stimulation in their relatives` everyday life more strongly than caregivers with lower education. This might cause a ceiling effect. In general, it is important for individuals with dementia to be provided with a stimulating environment because they often no longer have the abilities or opportunities to actively seek such stimulation on their own.

### Strengths and Limitations

Our study has several strengths. First, this is the first responder analysis for a non-pharmacological treatment in cognitively impaired persons. Second, we investigated patient-reported outcomes and personally relevant constructs, and cognition and ADL abilities were assessed via performance tests. Third, the sample size was sufficiently high with n = 362 individuals with cognitive impairment in day-care settings.

In health-services research, there is general agreement that response criteria with respect to the success of a therapeutic approach should not be based solely on expert ratings or characteristic parameters but should include patient-reported outcomes (“PROs”) (39). Researchers have suggested (40) that there is a need for research concerning the definition of patient-relevant endpoints that reflect the patient’s perspective on satisfaction with the care or treatment received (41). Research on PROs usually focuses on patients with physical impairments who are cognitively unaffected (42). However, for people with cognitive impairments, a reliable and valid self-rating is severely limited. Therefore, performance tests (e.g., in our study, the MMSE [cognition] and the ETAM [ADL]) have to be preferred.

On the other hand, our study has some limitations. First, the data referred only to the day-care setting, and thus, we do not know if the findings can be generalized to nursing home patients or individuals with dementia living in the community without formal support measures. Second, the MMSE is easy to administer and is often used in clinical studies, but it has a limited sensitivity to change. It would therefore be desirable to replicate our results using a more sensitive performance test for cognition. Third, there might be predictors or moderators of response that we could not find because we did not collect them (e.g., social network strength, neurophysiological markers, or Big Five personality factors). Fourth, it has to be noted, that 36.5% of the participants in our study did not have an “official” dementia diagnosis from a physician (but were classified by MMSE and MoCA). This might be due to recruitment of study participants in “normal” day-care settings (where a dementia diagnosis is not necessary and might sometimes not be transferred from the patient charts to the day-care patient charts) and not in specialized memory clinics or memory consultation centers. Furthermore, there are no incentives for physicians to diagnose dementia and no obligatory nationwide epidemiological registries to record these data in Germany.

## Conclusion

The response rates from MAKS therapy were the same in size or higher than those reported for anti-dementia drugs. There were only a few differences between responders and non-responders on the response criteria cognition, activities of daily living, and behavioral and psychological symptoms of dementia. Furthermore, these differences were of limited value for prediction because the rate of correct classifications was low. Responses to these non-pharmacological interventions did not seem to be predictable. Thus, MAKS therapy seemed to be suitable to the same degree for all participants included in this study—individuals with mild cognitive impairment, mild dementia, or moderate dementia. No special selection criteria are needed for the application of MAKS therapy in day-care facilities.

## Data Availability

The datasets generated for this study are available on request to the corresponding author.

## Ethics Statement

This study was carried out in accordance with the recommendations of the Ethics Committee of the Friedrich-Alexander-University Erlangen-Nuremberg (Ref. 170_14 B) with written informed consent from all subjects. All subjects gave written informed consent in accordance with the Declaration of Helsinki. The protocol was approved by the Ethics Committee of the Friedrich-Alexander-University Erlangen-Nuremberg (Ref. 170_14 B).

## Author Contributions

KL, EG, and E–MB contributed substantially to the conception and design of the work and the acquisition of data for the work. KL, EG, E–MB, DÖ, CD, and JS contributed substantially to the analysis and interpretation of data for the work. KL, EG, JS drafted the work. KL, EG, E–MB, DÖ, CD, and JS revised the work critically for important intellectual content. All authors read and approved the final version of the manuscript. All authors agree to be accountable for all aspects of the work.

## Funding

This study was supported by grants from the German National Association of the Statutory Health Insurance and Long-Term Care Insurance Funds (GKV-Spitzenverband, Germany) (funding 92% of project costs), and the Bavarian State Ministry of Health and Care (Germany) (funding 8% of project costs).

## Conflict of Interest Statement

The authors declare that the research was conducted in the absence of any commercial or financial relationships that could be construed as a potential conflict of interest.

## Abbreviations

ADL, Activities of daily living; BIZA-D, Berliner Inventar zur Angehörigenbelastung – Demenz; BPSD, Behavioral and Psychological Symptoms of Dementia; BSFC-s, Burden Scale for Family Caregivers—short version; CEI, Cholinesterase inhibitor; CG, Control group; ETAM, Erlangen Test of Activities of Daily Living in Persons with Mild Dementia or Mild Cognitive Impairment; IG, Intervention group; MMSE, Mini-Mental State Examination; NPI-Q, Neuropsychiatric Inventory Questionnaire; NTT, Number needed to treat; PRO, Patient-reported outcome; PWD, Person with dementia.
